# Genetic Imaging of Neuroinflammation in Parkinson’s Disease: Recent Advancements

**DOI:** 10.3389/fcell.2021.655819

**Published:** 2021-07-15

**Authors:** Longping Yao, Jiayu Wu, Sumeyye Koc, Guohui Lu

**Affiliations:** ^1^Department of Neurosurgery, The First Affiliated Hospital of Nanchang University, Nanchang, China; ^2^Department of Neuroscience, Institute of Health Sciences, Ondokuz Mayıs University, Samsun, Turkey

**Keywords:** Parkinson’s disease, microglia, genetics, neuroinflamamation, dopaminergic neurons, neurotoxins

## Abstract

Parkinson’s disease (PD) is one of the most prevalent neurodegenerative aging disorders characterized by motor and non-motor symptoms due to the selective loss of midbrain dopaminergic (DA) neurons. The decreased viability of DA neurons slowly results in the appearance of motor symptoms such as rigidity, bradykinesia, resting tremor, and postural instability. These symptoms largely depend on DA nigrostriatal denervation. Pharmacological and surgical interventions are the main treatment for improving clinical symptoms, but it has not been possible to cure PD. Furthermore, the cause of neurodegeneration remains unclear. One of the possible neurodegeneration mechanisms is a chronic inflammation of the central nervous system, which is mediated by microglial cells. Impaired or dead DA neurons can directly lead to microglia activation, producing a large number of reactive oxygen species and pro-inflammatory cytokines. These cytotoxic factors contribute to the apoptosis and death of DA neurons, and the pathological process of neuroinflammation aggravates the primary morbid process and exacerbates ongoing neurodegeneration. Therefore, anti-inflammatory treatment exerts a robust neuroprotective effect in a mouse model of PD. Since discovering the first mutation in the α-synuclein gene (SNCA), which can cause disease-causing, PD has involved many genes and loci such as LRRK2, Parkin, SNCA, and PINK1. In this article, we summarize the critical descriptions of the genetic factors involved in PD’s occurrence and development (such as LRRK2, SNCA, Parkin, PINK1, and inflammasome), and these factors play a crucial role in neuroinflammation. Regulation of these signaling pathways and molecular factors related to these genetic factors can vastly improve the neuroinflammation of PD.

## Introduction

Parkinson’s disease (PD) is an age-related neurodegenerative disease characterized by motor and non-motor symptoms (Brundin et al., 2018). It is the second most common neurodegenerative disorder after Alzheimer’s disease, and now, it has become a significant public health problem worldwide (Fan, 2020). One characteristic pathological change is the loss of midbrain dopaminergic (DA) neurons (Charvin et al., 2018). Parkinson’s disease is usually characterized by the deposition of protein aggregates containing α-synuclein (Lewy bodies) in multiple brain regions (Rey et al., 2018). The decreased viability of DA neurons slowly results in the appearance of motor symptoms, which largely depend on dopaminergic nigrostriatal denervation. With the progression of neurodegeneration and advancing disease, people with PD also experience sleep disturbances, fatigue, altered mood, cognitive changes, autonomic dysfunction, and pain (Schapira et al., 2017). These “non-motor” symptoms dominate the clinical picture and are the main determinants of quality of life. Besides, some endophenotypes dominated by non-motor symptoms also have been reported in recent studies (Ehgoetz Martens and Shine, 2018). Nowadays, pharmacological and surgical interventions are the main treatments for improving clinical symptoms. However, preventing or curing the disease is not possible at present (Sarrafchi et al., 2016).

Although various possible pathogenetic mechanisms have been proposed in recent years, the cause of neurodegeneration remains unknown. One possible mechanism is inflammation mediated by microglial cells (Yao et al., 2018). Emerging evidence indicates that sustained inflammatory stimulation plays a vital role in the degeneration of DA neurons and is a common feature in both human PD patients and animal models of PD (Ikeda-Matsuo et al., 2019). The neuroinflammatory response may also lead to a cascade of events leading to neuronal degeneration. Anti-inflammatory treatment showed beneficial effects in preventing neurodegeneration mediated by inflammatory damage (Singh et al., 2020). For example, ursolic acid acted as a therapeutic drug targeted for protecting from neuroinflammation-induced neurodegeneration (Rai et al., 2019; Singh et al., 2020; Zahra et al., 2020). Besides, the supplement of tyrosine hydroxylase in MPTP-intoxicated mice could protect dopaminergic neurons by suppressing neuroinflammation (Birla et al., 2019). Thus, regulating neuroinflammation is a therapeutic strategy by reducing oxidative stress (Rai et al., 2017; Yadav et al., 2017; Singh et al., 2018). In recent years, research has shown that many genetic factors are associated with neurodegeneration of the nigrostriatal DA, which includes apoptosis or death caused by neuroinflammation (Zhang et al., 2018). These factors are potential targets to interfere with the disease process in PD. This review discusses the details of genetic imaging of neuroinflammation in PD.

## Inflammation and Parkinson’s Disease

**FIGURE 1:**
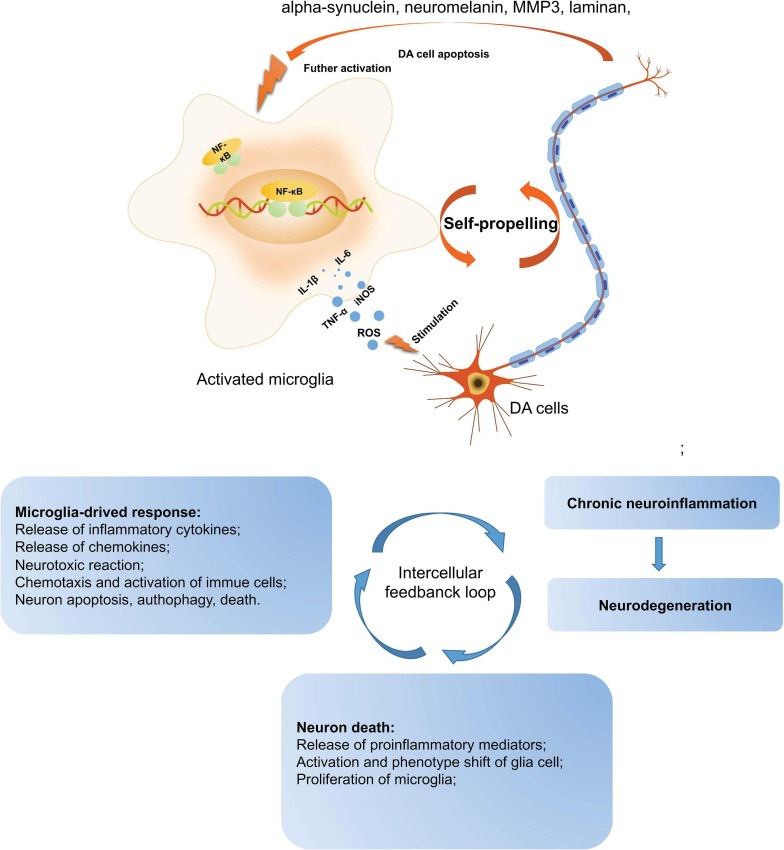
A self-propelled degeneration cycle in PD. In PD’s pathological conditions, microglia are activated and release anti-inflammatory cytokines to repair the tissues, protecting neurons against apoptosis or death. However, the continuous stimulation of pathological factors increases the number of toxic phenotypes of microglia, releasing a large number of inflammatory cytokines, such as TNF-α, IL-1β, iNOS, IL-6, and ROS, which contribute to neuronal damage. Besides, the impaired or dead DA neurons can directly induce microglial activation, increasing ROS and pro-inflammatory cytokines. Thus, the activation of microglia and DA neuronal damage form a self-propelled degeneration cycle in PD. PD, Parkinson’s disease; DA, dopaminergic; ROS, reactive oxygen species; iNOS, inducible nitric oxide synthase; IL-6, interleukin-6; TNF-α, tumor necrosis factor-α; IL-1β, interleukin-1β.

The presence of activated microglial cells in the substantia nigra has been shown in postmortem studies and in the 1-methyl-4-pheny-1, 2, 3, 6-tetrahydropyridine (MPTP)-animal models (both mice and non-human primates) of PD (Zella et al., 2019). Microglia are the macrophages that reside in the central nervous system (CNS) and are the brain’s primary innate immune effector cells (Yin et al., 2017). Besides, they are the main generator of inflammatory cytokines and reactive oxygen species (ROS), acting as the central active immune defense under normal conditions (Takeda et al., 2018). In pathological conditions, microglia are activated and releases anti-inflammatory cytokines and neurotrophic factors, contributing to tissue repair and the protection of neurons against apoptosis or death (Hilla et al., 2017). However, when pathological factors are continuously present, the number of noxious phenotypes of microglia increase and release a large amount of ROS, proteinases, and inflammatory cytokines contributing to neuronal damage. In PD, the inflammatory mediators such as tumor-α (TNF-α), interleukin-1β (IL-1β), inducible nitric oxide synthase (iNOS), and interleukin-6 (IL-6) have been found to regulate the progression of PD (Yao et al., 2019b). These pro-inflammatory mediators have been found to increase significantly in the midbrain of PD patients and animal models. Furthermore, numerous studies have shown that impaired or dead DA neurons can directly induce the activation of microglia, increasing the production of ROS and pro-inflammatory cytokines. Therefore, as mentioned above, the activation of microglia and DA neuronal damage form a self-propelled degeneration cycle in PD; thus, microglia are more likely to play critical roles in establishing and maintaining inflammatory responses in PD (Figure 1).

The anti-inflammatory treatment has been found to exert a strong neuroprotective effect in a mouse model of PD. For example, specially designed liposomes targeted for the CD163 receptor were loaded with glucocorticoids involved in PD-like neurodegeneration. The results showed that glucocorticoids protect DA neurons in the 6-hydroxydopamine (6-OHDA) PD model via anti-inflammatory modulation (Tentillier et al., 2016). APSE-Aq (fraction isolated from A. pyrifolium seeds) may have antioxidant and anti-inflammatory properties, which may offer neuroprotection in a model of PD (de Araujo et al., 2018). Tetramethylpyrazine exhibits its anti-apoptotic, anti-inflammatory, and antioxidant actions via attenuating rotenone-induced upregulation of the transcription factor nuclear factor erythroid 2-related factor 2/heme oxygenase-1 (Nrf2/HO-1) pathway and inflammation markers: nuclear factor-kappa B (NF-κB), iNOS, cyclooxygenase 2 (COX2), and glial fibrillary acidic protein (GFAP) expression (Lu et al., 2014; Michel et al., 2017). The isothiocyanate is mainly found in Brassica vegetables (Brassicaceae) and Moringaceae plants. It shows potent anti-inflammatory activity in the treatment of murine subacute PD and promises compounds against oxidative stress and neuroinflammation (Sita et al., 2016; Giacoppo et al., 2017). The neuroprotective properties of securinine may be due to the inhibition of glial activation and the subsequent generation of pro-inflammatory factors via inhibiting the p38 mitogen-activated protein kinase-NF-κB (MAPK-NF-κB) pathway (Leonoudakis et al., 2017). A novel compound (VSC2) has anti-inflammatory and antioxidant properties in microglia and in an animal model of PD by preventing NF-κB activation and the production of TNF-α, iNOS, IL-1β, NO, and COX-2 (Lee et al., 2015). Morin effectively prevents MPTP-induced PD-like pathologies in mice and protects primary neurons against MPP + −induced toxicity by ameliorating oxidative stress and inflammation (Zhang et al., 2010; Lee et al., 2016). The anti-inflammatory effect of β-Hydroxybutyric acid on microglia is mediated by G-protein-coupled receptor 109A. This process involves the NF-κB signaling pathway in both *in vivo* and *in vitro* PD models, causing the inhibition of the production of pro-inflammatory enzymes (iNOS and COX-2) and pro-inflammatory cytokines (TNF-α, IL-1β, and IL-6) (Fu et al., 2015). The synthesis and biological evaluation of clovamide analogs show that they have potent anti-neuroinflammatory effects by reducing the expression of GFAP in a model of PD (Hu et al., 2018). Compound 21 is obtained from 2,4,6-trimethoxybenzaldehyde by adding amines or alkyl (methyl or ethyl) ester of the amino acid hydrochloride salts. Compound 21 may be a potential candidate for PD treatment because of its potent anti-neuroinflammatory activity, novel mechanism, impressive penetration of the blood-brain barrier, and low toxicity *in vitro* and vivo (Wang et al., 2016). The behavioral and neurochemical alterations in a rat model of PD are partially reversed by Spirulina platensis, which is primarily related to its anti-inflammatory effects (Lima et al., 2017). Phytic acid shows a solid neuroprotective effect in an MPTP-induced PD model correlated with its anti-inflammatory effect by suppressing the NF-κB and phosphorylated extracellular signal-regulated kinase (p-ERK) pathways (Lv et al., 2015).

Based on the hypothesis that neuroinflammation is involved in the pathophysiology of PD, scientists have evaluated the feasibility of using non-steroidal anti-inflammatory drugs (NSAIDs) to cure PD patients. Interestingly, the results of a prospective cohort study showed that NSAIDs might delay or prevent the onset of PD (Chen et al., 2003). Ibuprofen users had a lower risk of PD than non-users, suggesting that ibuprofen use may delay or prevent PD onset (Poly et al., 2019). However, the same anti-inflammatory effect was not observed for aspirin, other NSAIDs, or acetaminophen in PD patients. The inconsistent results between ibuprofen and other NSAIDs indicate that ibuprofen has specific protective properties of the anti-inflammatory response in PD.

## The Role of NF-κB in Neuroinflammation in PD

Transcription factors such as NF-κB, STAT1, STAT3, and SMAD7 are the proteins that can bind to a specific sequence of DNA to regulate the transcription of genes. Recent literature shows that the upregulation of transcription factors can cause microglial activation, leading to a self-sustaining neuroinflammation environment via various mechanisms in PD. In this article, we will describe how NF-κB is associated with different pathways and molecular regulation. For now, we will give a clear description of the role of NF-κB in neuroinflammation in PD.

NF-κB is crucial for neuroinflammation responses; NF-κB is a group of transcription factors including RelA, RelB, c-Rel, NF-κB1/p50, and NF-κB2/p52 (Sokolova and Naumann, 2017). The transcription factors can form homodimers and heterodimers to regulate the expression of genes. The abundant basal expression of NF-κB in the brain is much higher than in peripheral tissues (Shih et al., 2015). NF-κB is largely involved in the progression of PD. For example, it reveals that NF-κB is activated in an MPTP model of PD (Dehmer et al., 2004), following the microglia activation (Aoki et al., 2009). One research study showed that NF-κB increased more than 70-fold in the brain tissue of PD patients and exhibited strong nuclear p65 immunoreactivity of DA neurons in the substantia nigra (Mattson and Camandola, 2001). The activated NF-κB leading to DA neuron degeneration has been demonstrated in PD (Phani et al., 2012). Selective inhibition NF-kB has been found to protect against DA neurons’ death from MPTP toxicity in a PD model (Bassani et al., 2015).

In neuroinflammation, NF-κB can regulate the production of pro-inflammatory cytokines, such as IL-6, TNF-α, G-CSF, iNOS, and IL-1β. The sustained inflammatory stimulus is related to the NF-κB pathway in the activation of uncontrolled microglia, resulting in ROS production, neurotoxic factors, interferon-γ (INF-γ), and glutamate, whose excessive formation induces neuronal damage (Spencer et al., 2012). Toll-like receptors are a vital class of membrane proteins that can activate microglial cells—the predominant pathways that TLRs trigger are associated with the NF-κB pathway (Kawai and Akira, 2007). Inhibition of the NF-κB pathway can sufficiently suppress the activation of microglia and neuroinflammation.

Recent studies show that NF-κB is involved in the inflammatory response of microglia in the progression of PD. Therefore, the regulation of the abnormal expression of NF-κB exerts fortissimo neuroprotection and inhibition of inflammation in PD. For example, our previous study found that miR-124 can prevent DA neuronal death and suppress microglia activation via suppressing the MEKK3/NF-κB pathway in a mouse model of PD (Yao et al., 2018). Intranasal plasma rich in growth factors (PRGF)-Endoret provides a novel neuroprotective strategy for DA and attenuates NF-κB-dependent inflammation processes in a PD model (Anitua et al., 2015). Inhibition of NF-kB activation leads to the suppression of pro-inflammatory molecules, improvement in locomotor activity, and DA neurons’ protection in the substantia nigra pars compacta (SNpc) (Mondal et al., 2012). In DA neuron-glial co-cultures, pioglitazone prevents DA cells’ death from lipopolysaccharide (LPS)-induced exacerbation of microglia activation by interfering with the NF-κB pathway (Esposito et al., 2007). Besides, schisandrol A could enhance the PI3K/AKT pathway and inhibit the IKK/IκBα/NF-κB pathway to reduce neuronal inflammation, oxidative stress and enhance the survival of DA neurons in the brains of PD mice (Yan et al., 2019). Rosmarinic acid could attenuate inflammatory responses by suppressing the HMGB1/TLR4/NF-κB signaling pathways, which may contribute to its anti-PD activity (Lv et al., 2019). Cordycepin mitigates MPTP-induced inflammatory response in PD by inhibiting the TLR/NF-κB signaling pathway (Cheng and Zhu, 2019). The knockdown of cathepsin D can protect dopaminergic neurons from neuroinflammation-mediated neurotoxicity via inhibition of the NF-κB signaling pathway in a PD model (Gan et al., 2018). Polydatin treatment protects DA neurons and ameliorates motor dysfunction by inhibiting microglial activation and the release of pro-inflammatory mediators via regulation of the AKT/GSK3β-Nrf2/NF-κB signaling axis (Huang et al., 2018).

Meanwhile, several NSAIDs, such as sodium salicylate, celecoxib, aspirin, and diclofenac, have been found to exert a neuroprotective role by decreasing NF-kB expression in an MPTP-induced model of PD (Bassani et al., 2015). The peroxisome proliferator-activated receptor γ (PPAR-γ) agonist pioglitazone mediates microglial activation and NF-κB expression in the 6-hydroxydopamine model of PD (Goes et al., 2018). A20 enzyme, which inhibits NF-κB by restricting the duration and intensity of its action, has been found to significantly decrease in a blood sample of patients with PD (Mazo et al., 2017). The topics which are discussed next are widely associated with NF-κB.

## The Role of Genetics in the Neuroinflammation of PD

Since discovering the first mutation in the α-synuclein gene (SNCA) can cause disease-causing, PD has involved many genes and loci. For example, the deficiencies of genes such as LRRK2, Parkin, SNCA, and PINK1 are risk factors for PD (including family and sporadic PD). The genetic discoveries clearly illustrate the cellular pathways and functions that are involved in the development of PD. To date, at least 23 loci and 19 genes (Table 1) have been identified and designated as both 10 autosomal dominant and 9 autosomal recessive PD.

**TABLE 1:** The genes associated with the pathogenesis of PD.

Gene	Full name	Locus	Location
SNCA	Synuclein alpha	PARK1	4q22.1 Azizi and Azizi (2018)
Parkin	Parkin RBR E3 ubiquitin protein ligase	PARK2	6q26 Morato Torres et al. (2020)
PARK3	Parkinson disease 3	PARK3	2p13 Kachidian and Gubellini (2021)
SNCA	Synuclein alpha	PARK4	4q22 Forero et al. (2020)
UCHL1	Ubiquitin C-terminal hydrolase L1	PARK5	4p13 Kachidian and Gubellini (2021)
PINK1	PTEN induced putative kinase 1	PARK6	1p36 Li D. et al. (2020)
PARK7	Parkinsonism associated deglycase	PARK7	1p36.23 Li Y. et al. (2020)
LRRK2	Leucine rich repeat kinase 2	PARK8	12q12 Li D. et al. (2020)
ATPase 13A2	ATPase 13A2	PARK9	1p36.13 Gago et al. (2020)
PARK10	Parkinson disease 10	PARK10	1p32 Kachidian and Gubellini (2021)
GIGYF2	GRB10 interacting GYF protein	PARK11	2q37.1 Calatayud et al. (2017)
PARK12	Parkinson disease 12	PARK12	Xq21-q25 Zanon (2020)
HTRA2	HtrA serine peptidase 2	PARK13	2p13.1 Zanon (2020)
PLA2G6	Phospholipase A2 group VI	PARK14	22q13.1 Sudira et al. (2018)
FBXO7	F-box protein 7	PARK15	22q12.3 Yuan et al. (2017)
PARK16	Parkinson disease 16	PARK16	1q32 Han et al. (2019)
VPS35	VPS35, retromer complex component	PARK17	16q11.2 Cutillo et al. (2020)
EIF4G1	Eukaryotic translation initiation factor 4 gamma 1	PARK18	3q27.1 Li D. et al. (2020)
DNAJC6	DnaJ heat shock protein family (Hsp40) member C6	PARK19	1p31.3 Velez-Pardo and Jimenez-Del-Rio (2020)
SYNJ1	Synaptojanin 1	PARK20	21q22.1 Zanon (2020)
TMEM230	Transmembrane protein 230	PARK21	20p13 Deng et al. (2018)
CHCHD2	Coiled-coil-helix-coiled-coil-helix domain containing 2	PARK22	7p11.2 Velez-Pardo and Jimenez-Del-Rio (2020)
VPS13C RIC3	Vacuolar protein sorting 13 homolog C acetylcholine receptor chaperone RIC3	PARK23	15q22.2 11p15.4 Zanon (2020)

Recent epidemiological and genetic studies have indicated that some PD-associated genes are involved in regulating neuroinflammation in the CNS. The discoveries of genetic factors highlight the biological mechanism of PD. According to the literature, we summarized whether the 19 genes associated with PD are also associated with neuroinflammation (Table 1). Understanding how genetic factors influence the inflammatory pathogenesis of PD can help decipher the disease’s etiology.

### Leucine-Rich Repeat Kinase 2 (LRRK2)

Missense mutations in the LRRK2 gene are the most common cause of autosomal-dominant inherited PD (Chan and Tan, 2017); the standard variants of the LRRK2 gene have also been associated with sporadic PD (Kluss et al., 2019). LRRK2 has been a therapeutic target for family and sporadic PD (Tufekci et al., 2012). The penetrance of LRRK2 mutations is incomplete in PD because the lifetime risk is estimated to be 22–32% in clinical populations, suggesting strong modifiers of LRRK2 disease (Goldwurm et al., 2007). Recently, genome-wide association studies show that LRRK2 is also involved in modifying immunogenic responses in PD (Moehle et al., 2012). Injecting LPS can strongly induce LRRK2 expression in SNpc in mice. Idiopathic PD patients and those with an LRRK2 mutation have increased levels of pro-inflammatory serum markers (Brockmann et al., 2016). However, no activated inflammatory profiles are observed in PD patients with a non-manifesting LRRK2 mutation (Brockmann et al., 2016). A recent study showed that the kinase activity of LRRK2 increased in microglia cells in sporadic PD postmortem tissue (Di Maio et al., 2018). Besides, the accumulation of α-syn results in increased ROS expression via inducing mitophagy in neurons, a process linked to LRRK2 activity (Choubey et al., 2011; Saez-Atienzar et al., 2014). LRRK2 can modulatesmokine (C–X3–C) receptor 1–mediated signaling pathways to modulate microglial activity (Ma et al., 2016). LRRK2 kinase activity contributes to neuroinflammation via phosphorylating p53 in PD, and the phosphorylation of p53 induces the expression of TNF-α (Muda et al., 2014).

LRRK2 is upstream of protein kinase A (PKA) and can negatively control PKA activity, thus modulating neuronal functions (Greggio et al., 2017). Meanwhile, it has been found that LRRK2 can control microglial inflammation by regulating PKA-mediated NF-κB p50 phosphorylation in microglia cells (Russo et al., 2015). The mutant variants of LRRK2 can vastly enhance the transcriptional activity of NF-κB in microglia (Kim et al., 2012). LRRK2 knockdown increases the levels of phosphorylated NF-κB p50 in primary microglial cells (Russo et al., 2015). Further, phosphorylated NF-κB translocates into the nucleus, competes with, and displaces DNA-bound p50:p50 to initiate mRNAs transcription (Zhong et al., 2002). Thus, LRRK2 may control its activation to affect the consequent transcription of pro-inflammatory mediators via NF-κB in microglia. A recent study demonstrated that LRRK2 acts as a negative regulator of the nuclear factor of activated T cell (NFAT) transcription factors which are associated with the inflammatory response in a large set of immune cells (Liu et al., 2011). Moreover, the abnormal activity of LRRK2 modulates the activation and phagocytosis of microglia cells by the hyperpolymerization of cytoskeleton components such as actin and β-tubulin (Russo et al., 2014). Moreover, LRRK2 suppresses focal adhesion kinase (FAK) Y397 phosphorylation through the phosphorylation of Thr–X–Arg/Lys (TXR) motif(s) in FAK in microglial cells (Choi et al., 2015). LRRK2 has a negative regulatory role in αSYN clearance through down-regulation of the endocytosis pathway in microglia (Maekawa et al., 2016). Besides, LRRK2 promotes mitochondrial alteration in microglia via Drp1 in a kinase-dependent manner, contributing to pro-inflammatory responses, which is regarded as a potential therapeutic target in PD (Ho et al., 2018). LRRK2 has been found to modulate neuroinflammation and neurotoxicity in models of human immunodeficiency virus 1-associated neurocognitive disorders (Puccini et al., 2015).

The inhibition of LRRK2 kinase activity attenuates the expression of pro-inflammatory microglial signaling to modulate neuroinflammation. In this context, several studies have identified that LRRK2 inhibitors show good physicochemical and pharmacokinetic properties and good selectivity and blood-brain barrier permeability against both kinases (Koshibu et al., 2015). Disruption of LRRK2 activity prevents a complete inflammatory response and microglial morphological remodeling (Moehle et al., 2012). As of now, some inhibitors of the LRRK2 gene have been found, showing a potential new neuroprotective role in PD (Lee et al., 2010). Manganese could induce neuroinflammation and the up-regulation of LRRK2 *in vitro* and *in vivo*, and the know down of LRRK2 can attenuate manganese-induced autophagy dysfunction and inflammation in microglia (Chen J. et al., 2018). Wave2, an actin-cytoskeletal regulator which can directly couple to LRRK2, mediates Lrrk2–G2019S-induced DA neuronal death in both macrophage-midbrain cocultures and *in vivo* in PD (Kim K. S. et al., 2018).

**FIGURE 2:**
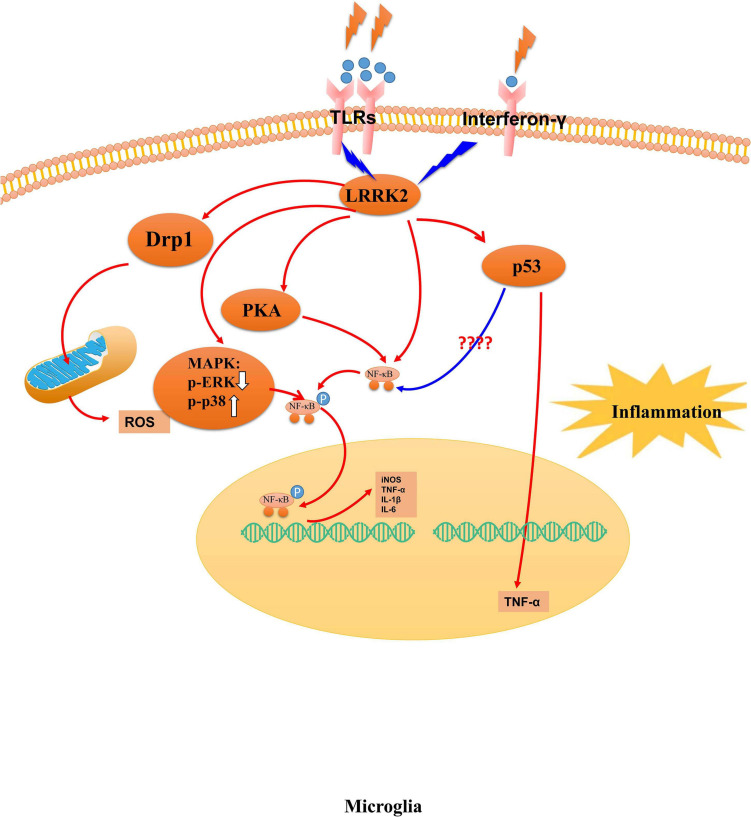
The mechanism of LRRK2 leading to microglia activation. Abnormal LRRK2 activity could regulate microglia cells’ activation through hyperphosphorylation of PKA, p53, MAPK family proteins, and Drp1. Thus, LRRK2 drives microglia toward a reactive phenotype with enhanced cell activity and inflammation in response to inflammatory stimuli, including LPS, environmental insults, and neuronal susceptibility.

Meanwhile, LRRK2 can phosphorylate Wave2 at the spot of Thr470, stabilize, and prevent its proteasomal degradation in a murine microglia-like cell line (Kim K. S. et al., 2018). The computer-aided drug design can prevent LPS-induced LRRK2 upregulation and microglia activation in a mouse model of neuroinflammation induced by LPS (Li et al., 2014). The overexpression of human pathogenic LRRK2 mutations exhibits long-term lipopolysaccharide-induced DA neuronal loss in mice, accompanied by exacerbated neuroinflammation in the brain (Kozina et al., 2018). Overall, LRRK2 is associated with the cellular pathways in microglia (Figure 2), and LRRK2 mutations with increased kinase activity might be one of the possible mechanisms for microglia- exacerbated neuroinflammation in PD.

### Alpha-Synuclein

**FIGURE 3:**
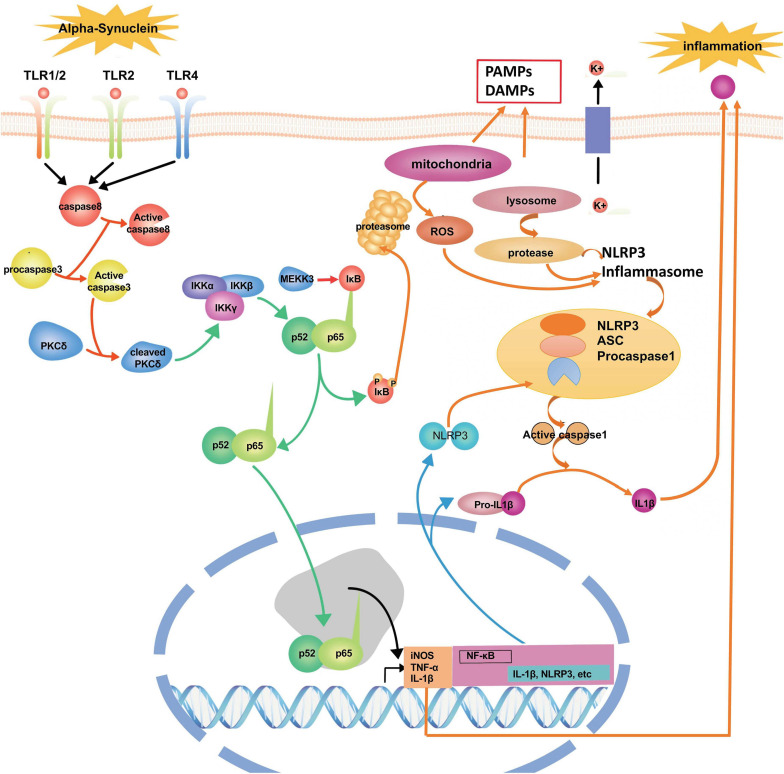
The signaling pathways and molecular factors involved in neuroinflammation. α-syn together with inflammasome form a network to regulate the activation of microglia. Blocking these signaling pathways and molecular factors can effectively improve apoptosis or the death of dopamine neurons caused by neuroinflammation.

Alpha-synuclein (α-syn), a 140-amino-acid protein, is abundantly expressed at a high level in the brain. Under physiological conditions, the functions of α-syn include the regulation of the dopamine transporter. The expression level of α-syn is regarded as a significant determinant of its neurotoxic potential. In contrast, secreted extracellular α-syn has emerged as an additional important factor in PD’s pathological process (Chen Y. et al., 2018). However, dysfunctional modifications of α-syn ultimately cause the pathogenesis of neurodegeneration in PD. In this pathological process, it has been shown that α-syn could trigger inflammation and oxidative stress through the activation of microglia (Harms et al., 2018). When the pathological deposition of α-syn occurs, microglia will migrate to the extracellular α-syn through endocytosis to prevent α-syn accumulation in neurons; however, the extensive uptake of α-syn with microglia could generate glial inclusions and induce inflammation (Vekrellis et al., 2011). Some *in vitro* and *in vivo* studies show that the release of α-syn from neurons can activate microglia through TLRs, followed by the initiation of neuroinflammation and progressive neuronal damage in PD (Zhang et al., 2005; Kim et al., 2013) (Figure 3). Besides, the deposition of α-syn in glial cells induces neuroinflammation, which promotes the degeneration of neurons and aggravates the pathogenesis of PD, and the deposition of α-syn also could further propagate to other glial cells and neurons (Chistiakov and Chistiakov, 2017). Specifically, the over-expression of α-syn could drive microglia into having a reactive phenotype characterized by enhanced levels of cytokine secretion, such as TNF-α and IL-6, as well as nitric oxide (NO), arachidonic acid metabolizing enzymes, and reactive nitrogen species, all superimposed upon impaired phagocytic potential (Rojanathammanee et al., 2011). Fibrillar α-syn, a potent inducer of pro-inflammatory immune, responds to microglia cells and highlights the level of fibrillization of α-syn as a significant feature for its efficient internalization and the activation process of microglia mainly depend on their aggregation state (Hoffmann et al., 2016). In the cerebrospinal fluid and blood of PD patients, researchers have found the aggregated and non-aggregated forms of α-syn. The type of secretion of α-syn into the medium has implied that this form of release from neurons may activate the inflammatory response in a microglial cell line (Alvarez-Erviti et al., 2011). Furthermore, α-syn deficiency promotes neuroinflammation by increasing Th1 cell-mediated immune responses. Endogenous α-syn plays a functional role in immunological processes during early experimental autoimmune encephalomyelitis (EAE) as a new regulator of Th1 responses in neuroinflammation (Ettle et al., 2016).

Dysfunctional modifications of α-syn affecting the activation of microglia are involved in many pathways in PD, and blocking these pathways can effectively control or attenuate neuroinflammation in PD. For instance, suppressing the Janus kinase/signal transducers and activators of transcription (JAK/STAT) pathway can prevent neuroinflammation and neurodegeneration by inhibiting microglial activation, macrophage, and CD4(+) T-cell infiltration, and the production of pro-inflammatory cytokines/chemokines induced by α-syn (Qin et al., 2016). Furthermore, six monosaccharides (6-mer), a specific inhibitor of the α-syn output, could efficiently modulate neuroinflammation and α-syn expression in neuron-like SH-SY5Y cells by blocking NF-κB activation (Scuruchi et al., 2016). Meanwhile, α-M inhibits α-syn-induced microglial neuroinflammation and neurotoxicity by targeting nicotinamide adenine dinucleotide phosphate (NADPH) oxidase as a therapeutic possibility in preventing PD progression (Hu et al., 2016). Furthermore, ginsenoside Rg1 could attenuate motor impairment and neuroinflammation in the MPTP-probenecid-induced PD mouse model via targeting α-syn abnormalities in the substantia nigra (Heng et al., 2016). FK506, as a well-known immunosuppressive drug, could decrease neuroinflammation and DA neurodegeneration, pointing to a causal role of neuroinflammation in an α-syn-based rat model of PD (Van der Perren et al., 2015). The administration of hypoestoxide could reduce neuroinflammation, neurodegeneration, and α-syn accumulation in a mouse model of PD via modulating the activity of NF-κB, suggesting that hypoestoxide may be a potent anti-PD drug (Kim et al., 2015). Curcumin has been found to afford its neuroprotective effect and inhibit α-syn aggregation through the inhibition of oxidative stress generation, replenishing glutathione levels, and preventing glial-associated inflammatory response in an LPS-induced PD model (Sharma et al., 2017; Sharma and Nehru, 2018). Endogenous high-mobility group protein B1, which has been demonstrated to mediate persistent neuroinflammation and consequent progressive neurodegeneration by promoting multiple inflammatory and neurotoxic factors, could promote the autophagic degradation α-syn via the Atg 5-dependent autophagy-initiation pathway in PD (Guan et al., 2018). Immunotherapy targeting TLR2 alleviates α-syn accumulation in neuronal and astroglial cells and attenuates neuroinflammation, neurodegeneration, and behavioral deficits in PD models of synucleinopathy by modulating α-syn transmission and neuroinflammation (Kim C. et al., 2018). Indeed, neuroinflammation might be mediated by the microglial expression of MHC II, a vital regulator of the immune response, given the central role for microglial MHCII in the activation of both innate and adaptive immune responses to α-syn in PD (Harms et al., 2013).

### The PINK1–Parkin Axis

#### Parkin

Parkin is predominantly expressed in the brain. It has been implicated in many biological processes, such as synaptic excitability, inflammation, and immunity. The protein comprises a C-terminal R1-in-between-ring-RING2 motif, an N-terminal ubiquitin-like (UBL) domain, RING0, RING1 IBR. In them, the Ubl and RING0 domains are unique to Parkin (Arkinson and Walden, 2018). The UBL domain interacts with the R1 environment, which negatively adjusts the activity of E3 ligase and parkin translocation to the mitochondria and parkin-dependent mitophagy. As an E3 ubiquitin-ligating enzyme, Parkin plays a critical role in the cell, which works together with E1 ubiquitin-activating enzymes and E2 ubiquitin-conjugating enzymes in a ubiquubiquitin-proteasomeem to ubiquitinate the misfolded or aggregated proteins (van der Merwe et al., 2015). Thus, Parkin can act as an activator to disrupt the autoinhibitory mechanisms and bridge the distance between catalytic sites. Parkin shows conformational changes after point mutation, disrupting the unique autoinhibitory features that release both REP and Ubl domain activity (Tang et al., 2017). Furthermore, the phosphorylation of Parkin leads to decreases in its E3 ubiquitin ligase activity (Aguirre et al., 2018). Besides, Parkin maintains mitochondrial quality control and turnover (McWilliams and Muqit, 2017).

The knockout of Parkin is a crucial way to study the role of Parkin in the pathogenesis of PD (Matheoud et al., 2019). The decrease in Parkin’s solubility and stability is associated with the degeneration of substantia nigra neurons in PD (Lonskaya et al., 2013). The MPTP treatment on mice increased the expression of Parkin and neuroinflammation (Mendes et al., 2019). Earlier studies reported the mutant Parkin in drosophila showed age-dependent degeneration of dorsomedial dopaminergic neurons (Cha et al., 2005; Whitworth et al., 2005). Recent studies have shown that Parkin’s S-nitrosylation could diminish its protective effects against α-synuclein-mediated neurotoxicity and destroy its ubiquitin ligase activity (Wahabi et al., 2018). It has been reported patients with mutated Parkin have clinical symptoms that are identical to patients with PD (Pickrell and Youle, 2015). Parkin mutation carriers are clinically characterized by slow disease progression and by having an excellent response to levodopa treatment. To date, more than 100 different mutations of Parkin have been reported, and the mutations are largely associated with the pathogenesis of PD (Abbas et al., 1999; Ferreira and Massano, 2017). The overexpression of Parkin could protect against manganese-induced cell death and dopaminergic toxicity (Higashi et al., 2004; Jiang et al., 2004).

Parkin shows a potential role in preventing neuroinflammation from progressing in PD. Mice with Parkin mutations appear to have selective DA neuron degeneration and some motor deficits with intraperitoneal LPS (Vivekanantham et al., 2015). Parkin levels and phenocopy Parkin loss-of-function mutations were found to be decreased in chronic inflammatory conditions, and the expression of TNF, IL-1β, and iNOS was increased in Parkin-null mice (Tran et al., 2011). In BV2 and primary microglia cell lines, the knockdown of Parkin was found to increase LPS-induced microglial activation by elevating the activity of NF-κB and JNK, which protected neurons from zVAD-mediated necroptosis (Mouton-Liger et al., 2018; Dionisio et al., 2019). Parkin deficiency could enhance NLRP3 inflammasome signaling by attenuating an A20-dependent negative feedback loop in mice (Mouton-Liger et al., 2018). The overexpression of thioredoxin reductase (TrxR) 2, a novel mediator of the inflammatory response, could efficiently alleviate inflammation-mediated neuronal death by activating the Akt–Parkin pathway and decreasing oxidative stress (Gao et al., 2019). TNF-α-mediated neuronal inflammation could be attenuated by mitochonic acid-5 via augmenting the AMPK-Sirt3 pathways and activating Parkin-related mitophagy (Huang et al., 2019).

#### PTEN-Induced Putative Kinase1 (PINK1)-Parkin Axis

Recent studies have highlighted that mitochondrial dysfunction and DNA abnormalities complicatedly associate with the pathogenesis of PD. PINK1 is a mitochondrial surveillance kinase that contributes to the processes involved in ridding the cell of damaged mitochondria; PINK1 mutations are a common genetic cause of familial PD. The mutations include truncating mutations, point mutations, missense, and deletions. PINK1-associated PD has an earlier age of onset and slower progression (Kawajiri et al., 2011). The examination of humans’ brains with PINK1-linked PD shows the pathology of Lewy bodies and neuronal loss in the substantia nigra, which are accompanied by microgliosis and astrocytic gliosis (Steele et al., 2015).

The PINK1 protein sequence contains a predicted C-terminal kinase domain and a mitochondrial targeting sequence of the N-terminus (Pickrell and Youle, 2015). The protein is imported into the mitochondria via the translocase of outer and inner membrane complexes. PINK1 controls mitochondrial quality control by removing the dysfunctional mitochondria. Mitochondrial damage is a significant cause of DA death in PD patients. PINK1 recruits the Parkin protein at the outer mitochondrial membrane while the damage of mitochondria occurred (van der Merwe et al., 2017). The activation of Parkin catalyzes the ubiquitination of outer mitochondrial membrane proteins with ubiquitin which is then degraded by the ubiquitin-proteasome system (Cornelissen et al., 2018). Autophagosomes engulf the dysfunctional organelles, and then lysosomal enzymes typically digest both of them via the process of mitophagy. PINK1/parkin-mediated mitophagy has been found in various neuronal and non-neuronal cells, especially exposing to mitochondrial depolarizing agents (Akabane et al., 2016; Newman and Shadel, 2018). The process prevents the accumulation of products from dysfunctional mitochondria, such as increased ROS and mtDNA damage (Truban et al., 2017).

**FIGURE 4:**
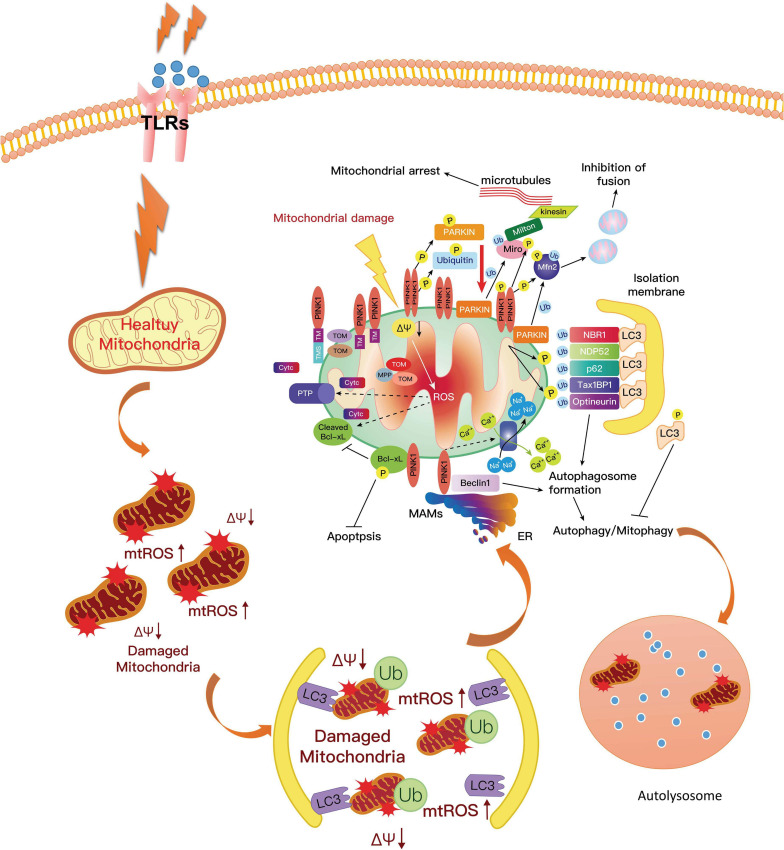
PINK1 could phosphorylate Parkin on the mitochondrial surface, resulting in the activation of Parkin. The activated Parkin proteins form phospho-polyubiquitin chains on damaged mitochondria. Finally, the dysfunctional mitochondria are cleared via autophagy. In this way, mitophagy inhibits neuroinflammation in PD and increases microglial phagocytosis. However, the mutation of PINK1 or Parkin would alter the balance of fission to fusion by preventing cells from responding to mitochondrial damage. The expression of ROS and pro-inflammatory factors are increased, which aggravates the development of PD. PD, Parkinson’s disease; ROS, reactive oxygen species.

In a poor state of PINK1, the prevention of accumulated products from dysfunctional mitochondria was obstructed, and mtDNA mutational stress resulted in an inflammatory response and activated the DNA-sensing cGAS–STING pathway, which connected mitoflammation with PD pathology (Sliter et al., 2018). In this study, the researchers also found that the expression of multiple cytokines such as IL-6, -12, and -13; IFNβ; CXCL1; and CCL2 and 4 increased efficiently in *Pink1^–/–^* and *Parkin^–/–^* mice. Meanwhile, it could also promote the expression of pro-inflammatory type-I IFN and inflammatory cytokine production and activate NF-κB signaling (West et al., 2015; West and Shadel, 2017). Furthermore, the mtRNA and their double-stranded mitochondrial RNA activated the inflammasome and the RNA-sensing immune receptor MDA5 (Dhir et al., 2018; Zhong et al., 2018). Moreover, one recent study by Yao et al. (2019a) reported that hydrogen-rich saline alleviated the inflammatory response and apoptosis via PINK1/Parkin-mediated mitophagy (Figure 4).

### Inflammasome

Inflammasomes, the central protein (varies with the type of inflammasome), which on activation recruits the adaptor apoptosis speck-like protein (ASC), is multimeric complexes consisted of a central protein, an adaptor protein ASC and a caspase-1 protein, forming in response to a variety of physiological and pathogenic stimuli. Inflammasome activation, accrued in both health and disease in the CNS, is an essential component of the innate immune. However, excessive activation of the inflammasome is also a significant driver of autoimmune and metabolic disorders, underlying the importance of understanding these physiological and pathological contexts (Sharma and Kanneganti, 2016; Mamik and Power, 2017). Recent work has mainly focused on the existence of inflammasome-mediated inflammatory pathways in CNS disorders. Pattern recognition receptors (PRRs) which are primarily expressed by glial cells, play an integral role in the innate immune response through the recognition of pathogen-specific proteins (PAMPs) and damage-associated proteins (DAMPs) (Singhal et al., 2014). So far, researchers have characterized four different inflammasomes and their activators, including NLRP1, NLRP2, NLRP3, nod-like receptor family CARD domain-containing protein 4 (NLRC4), and absent in melanoma 2 (AIM2) (Mariathasan et al., 2004; Boyden and Dietrich, 2006; Mariathasan et al., 2006; Rathinam et al., 2010; de Rivero Vaccari et al., 2014). Pattern recognition receptors have three distinguishing features: universal expression, fast response, and recognizing many microbes (Zhu et al., 2018). Based on these above features, PRRs efficiently initiate the signaling pathways culminating in the activation of MAPK, NF-κB, and interferon regulatory factors (IRFs), which control the transcription of genes encoding pro-inflammatory factors (Zhu et al., 2018). In neuroinflammation, inflammasomes can regulate microglial activation and subsequent neuroinflammatory processes in brain pathology (Scholz and Eder, 2017). Otherwise, α-syn enters into BV2 cells in an endocytosis-dependent manner and subsequently triggers NLRP3 inflammasome activation via inducing lysosomal swelling and increasing cathepsin B release (Zhou et al., 2016).

**FIGURE 5:**
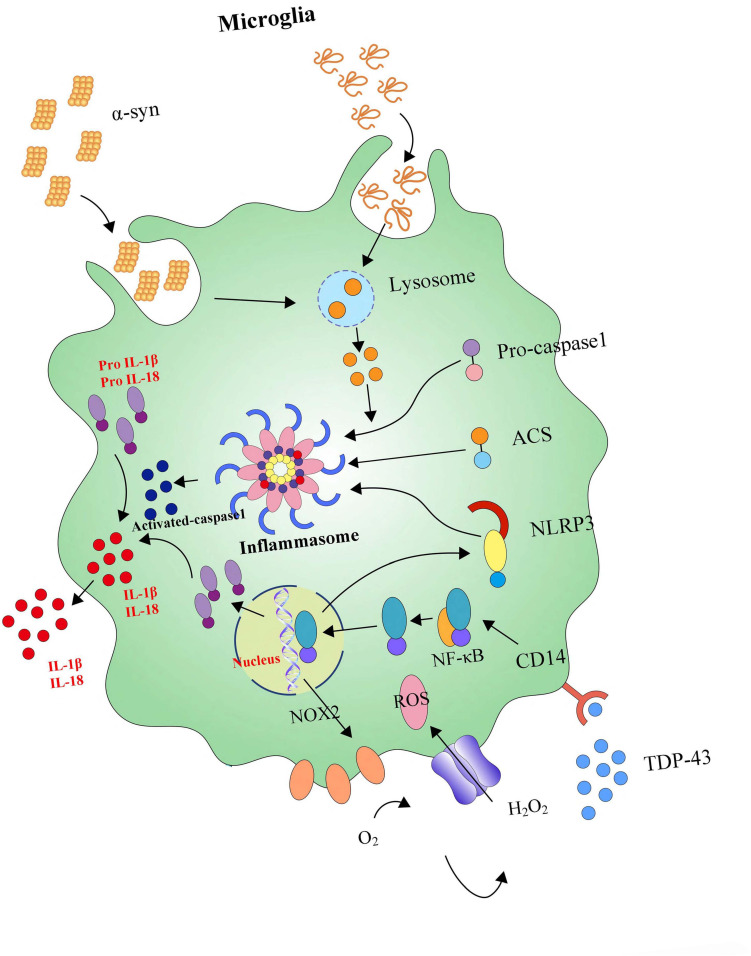
NLRP3 inflammasome activates neuroinflammation. Microglia are equipped with intracellular multi-molecule NLRP3 complexes, which α-syn can activate. NLRP3 inflammasomes could trigger the maturation of IL-1β and IL-18. High levels of IL-1β and IL-18 secretion enhances neuronal loss.

Meanwhile, it also has been found that inflammasomes cause caspase-1 activation following the stimulation of microglia with lysophosphatidylcholine (LPC), depending on LPS prestimulation, NLRP3, and adaptor ASC, and knockdown of inflammasome NLRC4 inhibits LPC-stimulated caspase-1 activity in microglia (Figure 5) (Scholz and Eder, 2017). Further, inflammasomes are also involved in the inflammatory pathogenesis of PD. For example, β-hydroxy butyrate (BHB), an effective inhibitor of the NLRP3 inflammasome in response to multiple activation stimuli including adenosine triphosphate (ATP), silica, and monosodium urate (MSU) crystals, almost completely blocks all aspects of inflammasome activation and pyroptosis induced by ATP and MSU crystals in PD (Youm et al., 2015; Deora et al., 2017). Crucially, modern studies suggest that the NLRP3 inflammasome could be a major disease-modifying therapeutic target in PD’s inflammatory pathogenesis. For instance, miR-7 directly targets NLRP3 expression (besides α-syn) and modulates NLRP3 inflammasome activation to attenuate DA neuronal degeneration accompanied by the amelioration of microglial activation in an MPTP-induced mouse model of PD (Zhou et al., 2016). In the heightened microglial activation response, an exaggerated ROS/c-Abelson murine leukemia viral oncogene homolog (c-Abl)/NLRP3 signaling axis evaluates in LPS-primed rotenone (ROT)-stimulated microglial cells and suggests that targeting c-Abl-regulated NLRP3 inflammasome signaling offers a novel therapeutic strategy for PD treatment (Lawana et al., 2017). Parkin deficiency modulates NLRP3 inflammasome activation by attenuating an A20-dependent negative feedback loop in Parkin’s pathogenesis (PARK2)-linked PD, paving the way for the exploration of its potential as a biomarker and treatment target (Mouton-Liger et al., 2018).

## Conclusion

Chronic inflammation of the CNS is mediated by neuroimmune microglial cells and has been implicated as a pathological contributor to PD. The activation of microglia and DA neuronal damage form a self-propelled degeneration cycle in PD; thus, microglia are more likely to play critical roles in establishing and maintaining inflammatory responses in PD. Currently, the signaling pathways and molecular factors involved in neuroinflammation have become an important research method to identify PD’s pathogenesis. The anti-inflammatory treatment has been found to exert a robust neuroprotective effect in a mouse model of PD. Studies on animal and cell models of PD have shown that dietary supplements containing polyphenolic compounds have beneficial effects and are recommended for treating and preventing inflammation-mediated neurodegeneration of DA neurons (Singh et al., 2020).

Studies have shown that blocking these signaling pathways and molecular factors can effectively improve apoptosis or the death of dopamine neurons caused by neuroinflammation. This paradigm is being shifted from theory to reality as a potential target for developing new drugs for PD. Going forward, focusing on these signaling pathways and molecular factors involved in neuroinflammation would provide a better understanding of the occurrence and development of PD. Ongoing research in this field may open a new door for developing pharmacological strategies toward the prevention and modification of the pathogenesis of PD.

## Author Contributions

LY and JW performed the majority of the literature search and predominantly contributed to the writing of the article. SK and GL assisted with the literature search. All authors read and approved the final manuscript.

## Conflict of Interest

The authors declare that the research was conducted in the absence of any commercial or financial relationships that could be construed as a potential conflict of interest. The handling editor is currently organizing a Research Topic with one of the authors GL.

## Abbreviations

6-mer: Six monosaccharides
6-OHDA: 6-hydroxydopamine
AIM2: Absent in melanoma 2
ATP: Adenosine triphosphate
BHB: β-hydroxy butyrate
c-Abl: c-Abelson murine leukemia viral oncogene homolog
CNS: Central nervous system
COX2: Cyclooxygenase 2
DA: Dopaminergic
DAMPs: damage associated proteins
EAE: Experimental autoimmune encephalomyelitis
FAK: Focal adhesion kinase
GFAP: Glial fibrillary acidic protein
IL-1 β: Interleukin-1 β
IL-6: Interleukin-6
iNOS: Inducible nitric oxide synthase
IRFs: Interferon regulatory factors
JAK/STAT: Janus kinase/signal transducers and activators of transcription
LPC: Lysophosphatidylcholine
LPS: Lipopolysaccharide
LRRK2: Leucine-rich repeat kinase 2
MAPK-NF- κ B: p38 mitogen-activated protein kinase-NF- κ B
MPTP, 1-methyl-4-pheny-1, 2, 3: 6-tetrahydropyridine
MSU: Monosodium urate
NADPH: Nicotinamide adenine dinucleotide phosphate
NFA: Nuclear factor of activated T cell
NF- κ B: Nuclear factor-kappa B
NLRC4: Nod-like receptor family CARD domain containing protein 4
NO: Nitric oxide
Nrf2/HO-1: Nuclear factor erythroid 2-related factor 2/heme oxygenase-1
PAMPs: Pathogen specific proteins
PD: Parkinson’s disease
p-ERK: Phosphorylated extracellular signal-regulated kinase
PINK1: PTEN-induced putative kinase 1
PKA: Protein kinase A
PPAR- γ: Peroxisome proliferator-activated receptor γ
PRGF: Plasma rich in growth factors
PRRs: Pattern recognition receptors
ROS: Reactive oxygen species
ROT: LPS-primed rotenone
SNCA: α-synuclein gene
SNpc: Substantia nigra pars compacta
TNF- α: Tumor necrosis factor- α
TrxR: Thioredoxin reductase
UBL: Ubiquitin-like
α-syn: Alpha-synuclein.
